# New Appliances and Things Medical

**Published:** 1898-05-07

**Authors:** 


### NEW APPLIANCES AND THINGS MEDICAL.

[We shall be glad to receive, at our Office, 28 & 29, Southampton Street, Strand, London, W.C., from the manufacturers, specimens of all new preparations and appliances which may be brought out from time to time.]

### DIABETIC GLYCERINE JUJUBES.

(Callard and Co., 65, Regent Street, W.)

We have received from Callard and Co. a sample of the above jujubes. They are exceedingly pleasant to the palate and of delicate flavour, and, being free from starch or sugar in any form, they may be safely given to diabetic patients. For the diabetic, who by the nature of his disease is cut off from most sweetmeats and delicacies of this nature, they will prove a considerable boon.

### CAMPHYLENE DISINFECTANTS.

(Fleming's Oil and Chemical Company 101, Leadenhall Street, London, E.C.)

Camphylene as a disinfectant and deodoriser is, perhaps, one of the most useful mediums available for domestic and general use. Owing to the energy and resource of Messrs. Fleming and Co. it may now be obtained in a variety of forms specially adapted for the multitudinous purposes to which disinfectants are now put. In the solid form it is prepared in large shields or in smaller blocks, which may be respectively hung up or otherwise placed in closets, lavatories, or sick-rooms. By a process of slow evaporation, the disinfectant is imparted to the surrounding air on which it exercises its disinfectant action. In powder form it may be used as an insect or beetle destroyer. In the fluid form, which is non-poisonous, it may be used as a general disinfectant and germicide for disinfecting wearing apparel, rooms, closets, &c. In fairly concentrated solution it appears to be a most deadly germicide, destroying pathogenic bacteria in a few seconds, and even when extensively diluted (5 per cent.) if left in contact with them for some 60 minutes it is equally efficacious. For the disinfection of typhoid, cholera, and other dangerous evacuations it has an obvious use. A more elegant preparation for domestic purposes is the ammoniated cream, which is a refreshing addition to the bath, exercising an invigorating as well as a disinfectant action on the skin; it may also be used for removing grease and other stains from cloths, carpets, curtains, &c.; and in this connection it should be remembered that for disinfecting these articles after exposure to infection in sick-rooms it is particularly useful. The Mikado moth paper will be recognised as an efficient preventive for moths in cloth, rugs, carpets, &c. The sheets should be placed between the folds of the article it is desired to protect.

### TOILET AND ANTISEPIC SOAPS.

(Edward Cook and Co., London.)

We have before had occasion to mention the various soaps emanating from the above firm, and to draw attention to the purity of their composition and the care with which they are manufactured. Cook's antiseptic soap is particularly commended to the notice of medical men, nurses, and public authorities who are responsible for the care and treatment of infectious diseases. The biniodide which is the active antiseptic agent in this soap is one of the most energetic and effective germicides known to science, and it has been incorporated with the soap in such a way as to render its antiseptic powers available for all purposes where soap and water can be applied. Thus for washing clothes, the skin of fever patients, unhealthy wounds, the instruments and hands of surgeons, we know of no more satisfactory medium. Other antiseptic soaps prepared by the same firm are coal tar, eucalyptous and the sulphur and glycerine varieties. All of them have their special application for skin diseases of diverse nature—the selection of any particular form should be left to the decision of the medical man.

For ordinary domestic use, the Riviera super-fatted soap is an agreeable and first-class preparation. As a toilet soap, it is specially suitable for delicate skins, owing to its great purity, absence of free alkali and colouring matter.

For the luxuriously inclined the Savon de Luxe is a toilet soap which cannot be beaten, not only for the delicacy of its scent, but also for the other features which are essential in such an article.

